# Spatial Distribution of Cosmetic-Procedure Businesses in Two U.S. Cities: A Pilot Mapping and Validation Study

**DOI:** 10.3390/ijerph10126832

**Published:** 2013-12-06

**Authors:** S. Bryn Austin, Allegra R. Gordon, Grace A. Kennedy, Kendrin R. Sonneville, Jeffrey Blossom, Emily A. Blood

**Affiliations:** 1 Division of Adolescent and Young Adult Medicine, Boston Children’s Hospital, 333 Longwood Ave., #634, Boston, MA 02115, USA; E-Mails: grace.kennedy@childrens.harvard.edu (G.A.K.); kendrin.sonneville@childrens.harvard.edu (K.R.S.); emily.blood@childrens.harvard.edu (E.A.B.); 2 Department of Social and Behavioral Sciences, Harvard School of Public Health, Boston, MA 02115, USA; E-Mail: argordon@mail.harvard.edu; 3 Department of Pediatrics, Harvard Medical School, Boston, MA 02115, USA; 4 Center for Geographic Analysis, Harvard University, Boston, MA 02115, USA; E-Mail: jblossom@cga.harvard.edu; 5 Clinical Research Center, Boston Children’s Hospital, Boston, MA 02115, USA

**Keywords:** cosmetic surgery, cosmetic procedure, U.V. indoor tanning, small-area estimation, NAICS code

## Abstract

Cosmetic procedures have proliferated rapidly over the past few decades, with over $11 billion spent on cosmetic surgeries and other minimally invasive procedures and another $2.9 billion spent on U.V. indoor tanning in 2012 in the United States alone. While research interest is increasing in tandem with the growth of the industry, methods have yet to be developed to identify and geographically locate the myriad types of businesses purveying cosmetic procedures. Geographic location of cosmetic-procedure businesses is a critical element in understanding the public health impact of this industry; however no studies we are aware of have developed valid and feasible methods for spatial analyses of these types of businesses. The aim of this pilot validation study was to establish the feasibility of identifying businesses offering surgical and minimally invasive cosmetic procedures and to characterize the spatial distribution of these businesses. We developed and tested three methods for creating a geocoded list of cosmetic-procedure businesses in Boston (MA) and Seattle (WA), USA, comparing each method on sensitivity and staff time required per confirmed cosmetic-procedure business. Methods varied substantially. Our findings represent an important step toward enabling rigorous health-linked spatial analyses of the health implications of this little-understood industry.

## 1. Introduction

### 1.1. Cosmetic Procedures: A Rapidly Proliferating Industry

Societal appearance ideals—whether to be unrealistically thin, muscular, tanned, or free of wrinkles or blemishes—place pervasive pressures on youth and adults to modify their appearance to avoid stigma associated with not conforming with perceived ideals [1,2,3,4,5,6]. While use of potentially harmful behaviors to control appearance, weight, and shape is more common in females than males, largely due to gender socialization processes that strongly align female worth with physical appearance [7,8], adolescents and adults of both genders, of all race/ethnicity and socioeconomic groups, and in developed and developing economies have been found to engage in these behaviors [9,10,11,12,13,14]. It is in this societal and historical context that industries that market services exclusively for cosmetic body modification—including surgical and minimally invasive procedures and ultraviolet (U.V.) indoor tanning—have recently experienced enormous increases in their customer base, volume of procedures conducted, and revenue. 

Over the past ten years cosmetic procedures as a whole have grown substantially in the United States. The number of cosmetic procedures performed in 2012 is almost double the number performed in 2000, with the American Society of Plastic Surgeons (ASPS) reporting that 14.6 million cosmetic procedures were performed by ASPS members and other surgeons in 2012, compared to 7.4 million in 2000 [15]. In addition, from 1997 to 2012, there has been an increase of over 250% in the total number of cosmetic procedures in the United States, with minimally invasive procedures increasing by 461% and cosmetic surgeries increasing by 80% [16]. The result is over $11 billion dollars spent on cosmetic procedures in 2012 in the United States [16]. Rates of cosmetic procedures are increasing in all race/ethnicity and age groups and in both women and men; however, the industry remains one essentially built on women, with 91% of people receiving cosmetic procedures in the United States being women [17].

U.V. indoor tanning is another highly popular procedure for cosmetic body modification in the United States. According to a 2012 industry report, an estimated $2.9 billion was spent annually in the country on U.V. indoor tanning [18]. Prior to the 2000s the tanning salon industry saw a steady incline in revenue; however, due to the economic recession of 2008 the industry experienced a decrease in revenue due to a lack of discretionary spending among consumers. Currently, the annual revenue of tanning salons is on the rebound and expected to reach its pre-recession profit levels by 2014 [18]. The U.S. Center for Disease Control estimates that nationwide 5.6% of American adults, or over 15 million people, used U.V. indoor tanning in the past 12 months [19]. Working with data from a recent National Cancer Institute study, Choi *et al.* found that more than a third of white U.S. women ages 18–24 years report use of indoor tanning beds in the past year [20], while another study of Florida high school students found white Latina/o youth were 2.5 times more likely than white non-Latina/o youth to have used indoor tanning in the past year [13]. 

### 1.2. Health Risks Associated with Cosmetic Procedures

Cosmetic procedures, which are distinguished from reconstructive procedures subsequent to illness, injury, or birth defect, are by definition not medically necessary and therefore cannot be considered to offer medical benefits to patients. However, they are associated with a wide range of health risks, as described below. There is very little regulation of cosmetic procedures by the U.S. federal government or in any U.S. state, which has contributed to performance of procedures by minimally trained practitioners, improperly conducted procedures, and inadequate infection control [21,22]. The majority of industry growth over the past decade has been in lower-cost minimally invasive procedures, such as subdermal injections of Botox^℘^, fat, and other substances. In the United States, any physician, regardless of specialty training, is permitted to perform cosmetic surgery and procedures [22]. 

The types of health risks to patients vary widely depending on the procedure and setting in which it is conducted. For instance, liquid silicone injections, which are used to change the shape of breasts, buttocks, and hips, can result in medical complications, even when medical grade silicone is used, when injected in excess quantity or by an inadequately trained provider. Injections of liquid silicone can cause life-threatening conditions, including acute renal failure and pulmonary embolism [23,24,25,26]. In addition, illegal injection of industrial grade liquid silicone and silicone adulterated by other materials, such as cement, have been documented throughout the country. The illegal injection market particularly targets low-income, immigrant, and transgender women [23,24,25,26,27].

Cosmetic breast implants have received decades of scrutiny from a variety of government regulatory agencies, and many complications have been documented [28]. The U.S. Institute of Medicine published in 1999 a report entitled Safety of Silicone Breast Implants, parsing out the research up to that point on “systemic” and “local” medical complications. The report indicated clear evidence of safety concerns with breast implants due to “local” complications, which include rupture, pain, capsular contracture, disfigurement, inability to breast feed, loss of sensation, and infection. These complications can require medical intervention and repeat surgeries [29]. Other health risks have been documented as well. For instance, breast implants may delay detection of breast cancer, potentially worsening prognosis [30,31]. It has been established for more than a decade that women who have cosmetic breast implants have two to three times the risk of suicide and other external causes of death, such as substance abuse and motor vehicle accidents, compared to women who have not undergone the procedure, likely related to elevated rates of preexisting psychopathology in women who seek cosmetic breast implants [32,33,34,35,36].

Breast implants are not lifetime devices, and it is likely that a woman who gets cosmetic implants in adolescence or young adulthood will have to have them surgically removed and replaced multiple times in her lifetime [37]. While cosmetic breast implants are not approved in the United States for adolescents younger than 18 years, U.S. Food and Drug Administration (FDA) nevertheless permits surgeons to implant the devices in minors as “off label” use [38]. FDA post-approval documents on breast implants report that, according to manufacturers’ own estimates, implants have at least a 20% failure rate within 10 years [29]. In addition, FDA documents report that two premarket approval studies conducted by industry have estimated reoperation rates to range between 28% and 44% in women within the first three years of implantation of the devices [39].

Liposuction and abdominoplasty (popularly known as “tummy tuck”) are procedures developed by surgeons and marketed specifically as ways to alter the effects of adiposity on appearance. There is no evidence that either procedure results in improvements in insulin action or metabolic risk factors for coronary heart disease [40,41], and evidence of any other beneficial health effects is lacking [42]. These procedures, however, are associated with high rates of morbidity and mortality, especially abdominoplasty [43,44,45]. Causes of death due to liposuction and abdominoplasty include pulmonary embolism, perforation, infection, and anesthesia reaction or overdose [43]. While a large scale, systematic study of fatality rates from a wide range of cosmetic procedures has not been done, Yoho and colleagues estimated fatality rates for select cosmetic procedures with comparisons to general U.S. mortality rates [43]. Their estimates, calculated as deaths/people exposed (*i.e.*, receiving a procedure), were as follows: cosmetic breast implants, between 1/1,500 and 1/6,000; liposuction, 1/5,224; abdominoplasty, 1/600; and facelift, 1/1,000. The authors compare these estimated fatality rates to U.S. rates for homicide (1/16,000) and traffic accidents (1/7,000). 

U.V. indoor tanning also has been linked with health risks, particularly melanoma [46,47,48,49]. Compared to nonusers, melanoma risk is more than doubled for people who use U.V. indoor tanning beds once a month or more often over a period of years, and starting use before age 35 years confers additional risk [46]. The incidence of melanoma has risen much more steeply in women than men in the United States since U.V. tanning became popular in the 1980s [47]. Among melanoma patients under age 30 years, the attributable risk associated with U.V. tanning bed use is estimated to be as high as 76% [46].

### 1.3. Geography of the Cosmetic-Procedure Industry vis a vis Theoretical Model of Disordered and Harmful Appearance-Control Behaviors

Thompson *et al.* have proposed the Tripartite Influence Model to understand social processes contributing to body dissatisfaction and engagement in potentially harmful appearance-control behaviors [6]. The Tripartite Influence Model suggests societal appearance values are transmitted and reinforced through three primary domains of social interaction: peers, family, and media. These domains can operate through positive reward for conforming to ideals and negatively via stigma and social rejection for deviating from appearance ideals [6]. The intensity with which societal appearance ideals are transmitted and reinforced is highly gendered, with the pressure on girls and women far greater and more pervasive than for boys and men [7,8]. That said, shifts in societal appearance ideals have been documented, showing increasing pressure on boys and men to strive to meet these ideals despite the improbability of achieving them and the risks of trying [50,51]. The degree to which an individual internalizes societal ideals of attractiveness and compares the appearance of one’s body to that of others have been found in observational and experimental studies to be two important processes in the pathway linking domains of social interaction (*i.e.*, interaction with peers, family, and media) identified in the Tripartite Influence Model to onset and maintenance of body dissatisfaction and engagement in potentially harmful appearance-control behaviors [5,51,52].

In parallel to research on the Tripartite Influence Model, which emerged from the psychological literature, other streams of research situated in the public health literature have highlighted the importance of an expanding investigation of environmental influences on body dissatisfaction and disordered and harmful appearance-control behaviors [53,54,55,56,57]. This perspective expands the media domain in the Tripartite Influence Model to include other important aspects of the environment through which societal appearance values are transmitted and reinforced. In addition to media, the model could include products and services used for appearance-control and businesses that sell these products and services, for example, diet pills and laxatives [53,58] and, most pertinent to the present study, cosmetic-procedure businesses [54,55,59]. In addition to the potential to transmit and reinforce societal appearance ideals, the availability of particular products and services in communities is an indicator of access. For instance, the presence of multiple businesses in a downtown neighborhood advertising on store fronts that they offer indoor tanning and Botox^®^ treatments signals to neighbors and passers-by that these procedures are normative and accessible. In the context of cosmetic procedures, once societal appearance values are transmitted, reinforced, and internalized, access is a crucial final link for engagement in the risk behavior. As a result, geographic location of cosmetic-procedure businesses is a critical element in understanding the public health impact of this industry. 

In recent years, important advances in health-linked geographic analyses have illuminated how high-burden public health problems, such as use of tobacco and alcohol and consumption of fast food, are affected by the density and spatial distribution of neighborhood businesses that sell these products [60,61,62,63,64]. Importantly, density and spatial distribution provide insight into which populations are most exposed and with what intensity to particular types of businesses of public health concern in the environments where they live and work. This research on the geography of risk exposures has identified clear leverage points for environmental and policy interventions to protect the public’s health. Similar research is critically needed to document the density and spatial distribution of businesses purveying cosmetic procedures and U.V. indoor tanning. Identifying communities that are highly exposed and tracking intensification of exposure in neighborhoods over time as these types of businesses proliferate will be essential to monitor potential harms resulting from cosmetic procedures and healthcare expenses as a consequence of these harms.

Despite the public health importance of documenting and mapping the locations of proliferating cosmetic-procedure businesses, substantial barriers exist to conducting this type of research. One, no centralized, uniform system exists to catalogue businesses offering cosmetic procedures. To carry out studies of spatial indicators, researchers must first have access to a database that includes the businesses of interest and their geographic coordinates. While certain sectors of the industry are captured through accreditation of office-based surgical centers and ambulatory health centers, these types of public records do not systematically distinguish those offering cosmetic *vs.* noncosmetic surgical and medical procedures [65]. Second, business types can be identified by North American Industrial Classification System (NAICS) codes, which are standardized codes representing a business’ major product category or service (http://www.census.gov/eos/www/naics). While the six-digit NAICS codes are considered by the U.S. federal government to be the standard by which to classify businesses, there is no single code or set of codes uniquely delineated for cosmetic procedures. Any one of potentially dozens of NAICS codes used by these businesses may also be used in greater proportions by businesses that do not offer these cosmetic services. Even indoor tanning, which has been assigned NAICS code #812199, shares this code category (Other Personal Care Services) with massage and tattoo parlors and a number of other business types. In addition, because codes are assigned according to primary service offered, an establishment that makes U.V. indoor tanning beds available to their customers but is not primarily a tanning salon—a phenomenon that is becoming increasingly common as U.V. indoor tanning and cosmetic procedures infiltrate into other business categories [66,67,68]—is likely to be listed under the code for their primary service, for instance as a fitness and recreational sports center (code #713940) or beauty salon (code #812112) and not the code for tanning salons. Similarly, minimally invasive cosmetic procedures are increasingly infiltrating not only a variety of healthcare settings such as dentistry and family practices [69,70,71], but also nonmedical business settings such as beauty salons and gyms [72,73,74,75].

Given the compelling need to advance research into the fast growing cosmetic-procedure industry, coupled with the methodological complexity of conducting spatial research in the current context, we designed a pilot study to address this need. The aim of our pilot study was to develop and validate novel methods to identify businesses offering cosmetic procedures, including U.V. indoor tanning, in order to quantify the number of businesses, characterize the types of procedures offered, and document the spatial distribution of businesses using geographic coordinates in two large U.S. cities. 

## 2. Methods

### 2.1. Classification of Cosmetic-Procedure Businesses

To determine what types of surgical and minimally invasive procedures qualify as cosmetic, we first conducted a review of the regulatory, industry, and medical literatures describing cosmetic procedures and associated health risks. To our knowledge, no universal classification system for cosmetic-procedure businesses has been established. For our pilot validation study, our classification system followed definitions promulgated in 2011 by the ASPS, the largest plastic surgery specialty organization in the world (http://www.plasticsurgery.org/Cosmetic-Procedures.html; see Appendix A). In addition, although outside the scope of the ASPS definitions, we included businesses offering U.V. indoor tanning due to both the cosmetic nature of the service and the well-established health risks posed by high U.V. radiation exposure [46,47,48,49]. Henceforth, we use the terms *cosmetic procedures* and *cosmetic-procedures industries* to encompass cosmetic surgeries, other minimally invasive procedures, and U.V. indoor tanning. For data description and presentation, we further subdivided procedures into four categories: cosmetic surgery; Botox^®^ or Dysport^®^ injections; other minimally invasive procedures; and U.V. indoor tanning. See Table 1.

**Table 1:** General categorization of cosmetic procedures eligible for inclusion in business identification and mapping validation study in Boston, MA, and Seattle, WA.

Category	Description
Cosmetic Surgery	Includes implants, lifts, liposuction, tucks, reshaping
Botox^®^/Dysport^®^ minimally invasive procedures	Includes any injection of Botulinum Toxin Type A (marketed as Botox^®^ or Dysport^®^).
Other minimally invasive procedures	Includes cellulite treatment, IPL, laser hair removal, laser skin resurfacing, laser treatment of leg veins, chemical peels, microdermabrasion, sclerotherapy, and injectable soft tissue fillers (e.g., collagen, Radiesse^®^, Juvederm^®^, Restylane^®^, Sculptra^®^, Artefill^®^).
U.V. tanning	Includes businesses offering U.V. tanning; spray tanning not included in validation study.

### 2.2. Data Sources

#### 2.2.1. Online Search Engines

Three online search engines were used to identify cosmetic-procedure businesses meeting inclusion criteria in Boston, and Seattle: Google.com, Yahoo.com, and Bing.com. Boston and Seattle were selected for this pilot study because they are large cities with comparable population sizes (both approximately 635,000 residents [76]) in divergent geographic regions of the United States. The results of the Google.com search served as the base list and the results from Yahoo.com or Bing.com were added only if they did not appear in the initial Google.com search. Businesses offering eligible cosmetic procedures were recorded in Excel, including full address, phone number, URL, and categories of cosmetic procedures offered. The benefits of these online data sources include ease of use, low-cost, and frequency with which data are updated. 

#### 2.2.2. ESRI Business Analyst Database

The ESRI Business Analyst database available for the year 2011 was used to identify possible cosmetic-procedure businesses located in Boston and Seattle. ESRI Business Analyst is compiled by infoGroup and contains approximately 12 million geocoded business locations within the United States. Business types can be identified in the database by NAICS codes. We used NAICS codes to identify business categories that were likely to include businesses that provided cosmetic procedures. Benefits of this data source include its comprehensiveness and the ease with which it can be used for geospatial analysis. 

#### 2.2.3. Business Identification and Confirmation Procedures

The first phase of the study aimed to test and compare multiple techniques for estimating the total number of businesses offering cosmetic procedures. We developed three methods to identify businesses offering cosmetic procedures: Method 1 used keyword searches of the Internet search engines described above to identify and confirm cosmetic-procedure businesses with websites; Methods 2 and 3 each used the ESRI Business Analyst database to identify candidate cosmetic-procedure businesses followed by a web and phone protocol to confirm businesses. 

#### 2.2.4. Method 1: Internet Search Engines for Business Identification and Confirmation

For Method 1, keyword search terms used to identify cosmetic-procedure businesses were determined through an initial pilot and review process and were used identically for the three search engines along with the city name. Final search terms used were: *medical spa*, *med spa*, *medspa*, *medispa*, *cosmetic surgery*, *plastic surgery*, *cosmetic dermatology*, *skin care*, *aesthetics*, e*sthetics*, *laser hair removal*, *botox*, and *tanning*. Search terms were entered into the search engine to obtain a list of candidate businesses. All unique websites on the first 10 pages of results—each of which listed 10 URLs—were checked for eligibility by geographic location (*i.e.*, address fell within Boston or Seattle city limits) and procedures listed. Confirmation that candidate businesses offered cosmetic procedures meeting our definition was done via the businesses’ websites. When necessary, the business was called to clarify cosmetic procedures listed online; in most cases, online listings were sufficiently clear for classification. Portals listing individual medical practitioners (who may practice at multiple sites) and businesses without websites were excluded. Although we restricted our search to the first 10 pages of results, we typically found that by page 8 results were redundant or ineligible.

#### 2.2.5. Methods 2 and 3: ESRI Business Analyst Database for Business Identification

For Method 2, we first selected 11 keywords (see Table 2) that were likely to be used in the names of cosmetic-procedure businesses. We then searched for these keywords in the ESRI database and used results to compile a list of candidate businesses for each city. 

**Table 2:** Business identification and validation: Percentage of candidate businesses identified by keyword or North American Industrial Classification System (NAICS ^a^) code that were confirmed as cosmetic-procedure business via confirmation procedures ^b^,by method, in Boston and Seattle, 2011–2012.

Method	Keyword or NAICS code	Number (n) of Candidate Businesses Identified by Keyword/NAICS Code		NAICS description	% of Candidate Businesses Confirmed	
		Boston	Seattle		Boston	Seattle
Method 2	COSMETIC	7	15		28.6	33.3
	DERMATOLOGY	7	6		42.9	66.7
	ESTHETIC	4	15		100.0	53.3
	LASER	8	8		62.5	62.5
	MED_SPA	1	1		0.0	100.0
	PLASTIC_SURGERY	3	3		100.0	100.0
	SKIN_CARE	22	14		63.6	38.5
	SKIN	18	51		47.1	47.1
	SPA	57	22		90.0	36.4
	TAN	20	3		100.0	33.3
	TANNING	1	15		42.1	80.0
Method 2-ext	BEAUT	30	30		3.3	0.0
	HAIR	30	30		3.3	6.7
	NAIL	30	30		3.3	16.7
	SALON	30	30		6.7	10.0
Method 3	446120	30	30	Cosmetics, beauty, supplies, perfume stores	10.0	10.0
	448190	NA	30	Other clothing stores	NA	3.3
	448310	^c^	NA	Jewelry stores	^c^	NA
	453998	NA	30	All other miscellaneous store retailers (excluding tobacco stores)	NA	3.3
	524114	NA	2	Direct health and medical insurance carriers	NA	0.0
	541614	NA	30	Process, physical distribution & logistics consulting services	NA	0.0
	611511	15	30	Cosmetology & barber schools	53.3	33.3
	621111	30	30	Offices of physicians (except mental health)	23.3	16.7
	621210	NA	30	Offices of dentists	NA	6.7
	621340	30	NA	Offices of physical, occupational, speech therapists	0.0	NA
	621493	30	NA	Freestanding ambulatory surgical & emergency centers	10.0	NA
	621498	8	13	All other outpatient care centers	37.5	46.2
	621999	30	NA	All other miscellaneous ambulatory health care services	0.0	NA
	713940	30	30	Fitness and recreational sports centers	3.3	3.3
	812112	30	30	Beauty salons	10.0	20.0
	812113	30	30	Nail salons	16.7	6.7
	812191	NA	30	Diet and weight reducing centers	NA	0.0
	812199	30	30	Other personal care (e.g., tanning, hair removal)	30.0	30.0
	812990	NA	^c^	All other personal services	NA	^c^
	813110	NA	^c^	Religious organizations	NA	^c^

Notes: ^a^ The 6-digit NAICS codes are considered the standard for U.S. federal agencies to classify businesses when collecting, analyzing, and publishing statistical data regarding the U.S. economy (http://www.census.gov/eos/www/naics/). ^b^ Percentage confirmed calculated as total number of confirmed businesses divided by total candidate businesses per keyword/NAICS code. ^c^ Excluded from verification due to implausibility of offering eligible services. NA: NAICS code not identified for this city via any of the validation methods.

An extension of this approach, Method 2-ext, added four additional keywords (see Table 2) to the original 11 keywords, thus increasing the number of candidate businesses. Lists of candidate businesses were then cleaned by review and removal of businesses that were captured in the keyword search for reasons unrelated to cosmetic procedures (e.g., for keyword *skin,* Ba*skin*-Robbins was removed; for keyword *laser*, G*laser*’s Towing was removed). This reduced the candidate list by 15% in Boston and 20% in Seattle. Businesses captured by multiple keywords (e.g., Seattle Skin & Laser was listed once for *skin* and once for *laser*) were included only once in the total number of candidate businesses but were included in the count for each keyword, as in Table 3.

**Table 3:** Business identification and validation: Distribution of cosmetic-procedure types among all confirmed businesses for each city and each method.

	**Method 1**			**Method 2**		
	**Boston (n = 60)**	**Seattle (n = 126)**	***p*^a^**	**Boston (n = 76)**	**Seattle (n = 64)**	***p*^a^**
Cosmetic Surgery	28.3%	20.6%	0.33	13.2%	12.5%	0.89
Botox^®^/Dysport^®^	40.0%	36.5%	0.77	21.1%	29.7%	0.33
Other Minimally Invasive Procedures	60.0%	77.0%	**0.03**	69.7%	73.4%	0.77
U.V. Tanning	41.7%	19.0%	**0.002**	30.3%	20.3%	0.25
	**Method 2 + 2-ext**			**Method 3**		
	**Boston (n = 81)**	**Seattle (n = 74)**	***p*^a^**	**Boston (n = 42)**	**Seattle (n = 46)**	***p*^a^**
Cosmetic Surgery	12.3%	10.8%	0.96	35.7%	23.9%	0.33
Botox^®^/Dysport^®^	19.8%	25.7%	0.49	40.5%	28.3%	0.33
Other Minimally Invasive Procedures	67.9%	77.0%	0.28	66.7%	63.0%	0.90
U.V. Tanning	33.3%	17.6%	**0.04**	26.2%	15.2%	0.31

Notes: Columns do not add to 100% because some businesses offered multiple categories of procedures.n: Number of businesses confirmed to provide eligible cosmetic procedures. ^a^ *P*-value from chi-square test of significant difference between Boston and Seattle in distribution of cosmetic-procedure businesses offering specified category of service.

For Method 3, we used NAICS codes found via the prior two methods. First, the NAICS codes associated with businesses identified as candidates by Method 1, 2, or 2-ext for each city was compiled. Second, all businesses with one of these NAICS codes in the 2011 ESRI database were extracted and considered to be candidate businesses. The composition of NAICS codes used to identify candidate businesses varied by city, possibly due to regional variations in the use of NAICS codes for cosmetic procedure-related businesses. Each business had only one NAICS code and therefore appeared only once in the final Method 3 list of candidate businesses.

#### 2.2.6. Methods 2 and 3: Business Confirmation

To appropriately classify candidate businesses identified via Methods 2, 2-ext, and 3, we conducted confirmation procedures for all or for a sample of candidate businesses in each city. Confirmation procedures were performed on all Method 2 candidate businesses. For Method 2-ext and Method 3, samples of 30 businesses per keyword or 30 businesses per NAICS code were drawn, respectively. One NAICS code identified in Boston (448310: Jewelry stores) and 2 in Seattle (812990: Other personal services, including escort services; and 813110: Religious organizations) were excluded from confirmation procedures due to the implausibility of offering eligible services. Confirmation procedures were conducted via website or phone. Websites alone were used for confirmation when a business’ website offered all necessary information to determine eligibility. For businesses that could not be confirmed via the Internet (52.2% in Boston and 42.8% in Seattle), a phone confirmation protocol was developed and implemented to ensure consistency and reliability of information collected. Businesses were called up to three times during standard business hours for the appropriate time zone. When a live person was reached, researchers followed a semi-structured phone-call script to inquire about all relevant procedures offered (see Appendix B); businesses for which a live staff person was never reached and businesses without working phone numbers were coded as missing. In Boston, 13.2% of candidate businesses were coded missing (77 unreachable businesses/582 candidates); in Seattle, 17.6% were coded as missing (110 unreachable businesses / 624 candidates). 

#### 2.2.7. Small-Areas Validation

The second phase of the study assessed the sensitivity of each of the three approaches to identifying cosmetic-procedure businesses. Assessing sensitivity required that we create a “gold-standard” list of businesses capturing all businesses offering cosmetic procedures within selected sub-areas within each city. For this phase of our study, we followed the following procedures: After mapping all businesses identified and confirmed by Methods 1, 2, 2-ext, or 3 in the first phase, we visually analyzed the distribution of businesses in each city and selected three sub-area polygons within each city, representing areas of equal size but varied density of cosmetic-procedure businesses (low, medium, and high). Small areas were uniform in size (about two-by-eight city blocks in size) and were classified as Low Density (one to two confirmed businesses), Medium Density (three to six), or High Density (seven or more). 

Once the three areas were selected, all candidate cosmetic-procedure businesses captured by Methods 2, 2-ext, or 3 within these sub-areas that were not already confirm during Phase 1 were then web-searched and, if necessary, called to confirm whether they offered relevant services. All confirmed cosmetic-procedure businesses were added to the gold-standard list in these sub-areas. Following this, we separately constructed lists of businesses that had originally been identified by Methods 1, 2, 2-ext, and/or 3 in these areas and sensitivity were calculated for each method. 

## 3. Analyses

### 3.1. Business Identification and Confirmation (First Phase)

We calculated the percentage of confirmed cosmetic-procedure businesses for each keyword used among all candidate businesses in Method 2 and for each keyword and each NAICS code among the subset of candidate businesses in which confirmation procedures had been carried out in the Method 2-ext and in Method 3. We also used chi-square tests to compare the distribution of types of cosmetic procedures identified in the two cities for each method. 

We used ArcGIS software to characterize the spatial distribution of selected cosmetic-procedure businesses within Boston and Seattle. The county, city, and census tract boundary datasets used in the analysis were downloaded from the U.S. Census Bureau website.

### 3.2. Small-Areas Validation (Second Phase)

Based on the small-areas validation study, we calculated sensitivity for each method. Sensitivity indicates the ability of a given method to accurately identify the true cases. For this study, sensitivity was calculated for each method as the proportion of businesses in a selected sub-area correctly identified as cosmetic businesses by that method out of all the cosmetic-procedure businesses in that sub-area (*i.e.*, the gold-standard list of all cosmetic businesses in that sub-area). For example, for Method 2 in the Boston High Density sub-area:

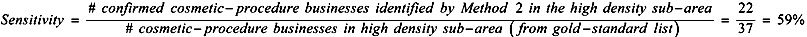


We also assessed the *staff time cost* as an additional important measure of efficiency using the small-areas validation study. This metric was calculated as the total minutes of staff time spent confirming whether or not the businesses identified by a method offered relevant cosmetic procedures divided by the total number of businesses identified by that method and confirmed as being cosmetic-procedure businesses. For example, for Method 3 in the Seattle Low Density sub-area:

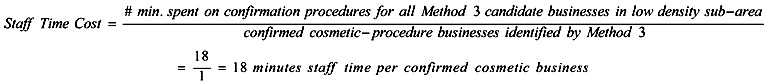


This process allowed us to compare the sensitivity and staff time cost of different methods, both independently and in combination with one another, with the aim of determining the most sensitive and time efficient approach to identifying cosmetic-procedure businesses for translation to larger-scale research.

## 4. Results

**Figure 1:**
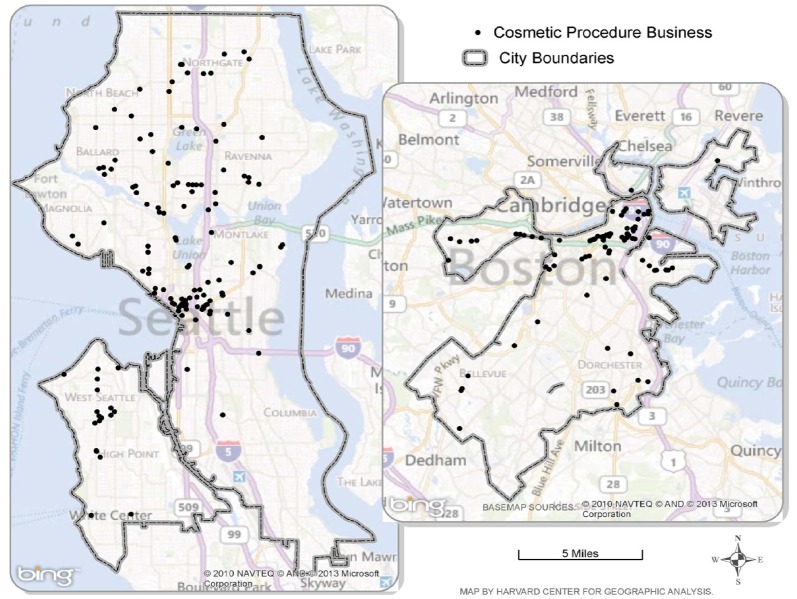
Boston and Seattle cosmetic-procedure businesses identified and confirmed via websearch (Method 1) and keyword search of ESRI Business Analyst database (Method 2), 2011–2012.

Note: Confirmation of candidate businesses was conducted using a web and phone protocol to determine whether each candidate offered services in any of four categories of cosmetic procedures (cosmetic surgery, Botox^®^/Dysport^®^, other minimally invasive procedures, and U.V. tanning).

Figure 1 depicts the spatial distribution of confirmed cosmetic-procedure businesses identified and confirmed by both web search (Method 1) and keyword search of the ESRI business database (Method 2) in Boston and Seattle, respectively. Methods 1 and 2 were selected for mapping as confirmation procedures on candidate businesses were carried out citywide for both methods, while confirmation procedures were carried out on only a sample of candidate businesses identified by Method 2-ext and Method 3 and thus were not assessments of the entire city. 

Table 2 presents the percentage of businesses that were confirmed as cosmetic-procedure businesses out of all those identified as candidates by each keyword or NAICS code used in Methods 2 and 3, respectively. The ability of an individual keyword to identify cosmetic-procedure businesses varied widely by keyword, as well as by city. For example, 80% of businesses identified using the keyword *spa* were confirmed as cosmetic-procedure businesses in Seattle, but only 42% were confirmed as such in Boston. The keyword *plastic_surgery* was the most successful keyword across both cities (100% of candidates were confirmed). Table 2 also demonstrates that the four keywords used for the Method 2-ext were not highly specific in identifying cosmetic-procedure businesses, with proportions of 10% or less of candidates across both cities being confirmed to offer eligible procedures. 

Among the NAICS codes used in Method 3, only one surpassed a 50% confirmation rate: NAICS code 611511, which is the code for cosmetology and barber schools. Of the businesses identified by this code, over half (53.3%) of those in Boston were confirmed as offering eligible cosmetic procedures, as were one-third (33.3%) of those in Seattle. Among businesses assigned the code for other outpatient care centers (621498), nearly half (46.2%) in Seattle and over one-third (37.5%) in Boston were confirmed to offer cosmetic procedures. 

Table 3 presents the distribution of types of cosmetic procedures that were offered among all businesses confirmed to offer any cosmetic procedures in each city. Across both cities, other minimally invasive procedures (e.g., chemical peels and microdermabrasion) were most commonly offered, with proportions of businesses offering these procedures ranging from 60.0% to 77.0%, depending on method.

As would be expected, cosmetic surgery was the least prevalent category of procedure offered—yet still was relatively common, estimated to be offered by 10.8%–35.7% of cosmetic-procedure businesses, depending on city and method. Notably, Method 1 and Method 3 identified a higher prevalence of cosmetic surgery businesses among all confirmed businesses compared to Method 2 and Method 2-ext (Boston: 28.3% and 35.7% compared to 13.2% and 12.3%, respectively; Seattle: 20.6% and 23.9% compared to 12.5% and 10.8%, respectively). With few exceptions, Boston and Seattle did not significantly differ in terms of the prevalence of each category of cosmetic procedure estimated by the different methods.

**Figure 2:**
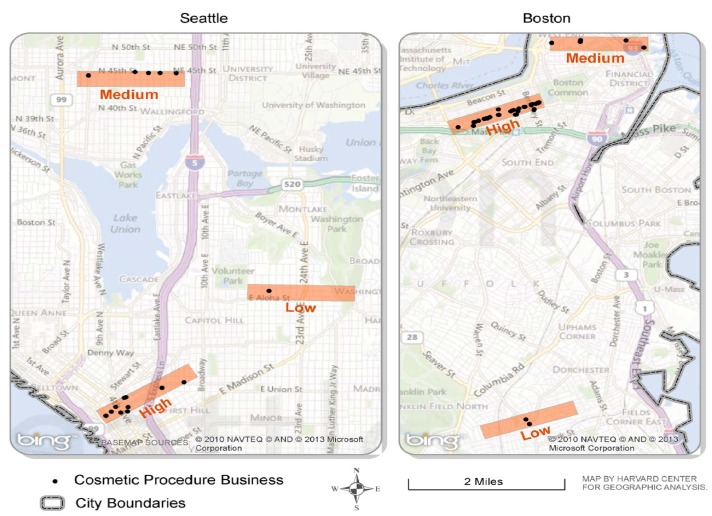
Small-areas validation: Boston and Seattle cosmetic-procedure businesses identified and confirmed in three sub-areas defined by density of confirmed cosmetic-procedure businesses citywide, 2011–2012.

Notes: Small areas were defined by density of cosmetic procedures businesses identified and confirmed via three methods: web-search (Method 1), keyword search of the ESRI Business Analyst database (Method 2), and NAICS code search of the ESRI database (Method 3). Small areas were uniform in size (1,508 × 260 meters) and were classified as Low Density (one to two confirmed businesses), Medium Density (three to six), or High Density (seven or more).

Figure 2 depicts each of the sub-areas (low, medium, and high density of cosmetic-procedure businesses) used in the small-areas validation study in Boston and Seattle, respectively. These maps also present the spatial distribution of cosmetic-procedure businesses that were identified and confirmed by any of the three methods in each sub-area. Key outcomes from the small-areas validation study are presented in Table 4. Sensitivity of each method, meaning its ability to identify the “true cases” of cosmetic-procedure businesses varied widely; sensitivity also varied by city and density of cosmetic businesses in the neighborhood. Comparing outcomes across all three sub-areas, Method 3 had the highest sensitivity (97.9% for Boston and 87.5% for Seattle), but was also the most resource-intensive method in terms of staff time cost (on average, 13 min were spent for every confirmed business for Boston and 18 min for Seattle). Combining methods, for example, Methods 1, 2, and 2-ext, led to somewhat higher sensitivities (75.0% for both cities), with a reduced staff time cost (7–8 min per confirmed business). Even more resource-efficient was a combination of Methods 1 and 2, without 2-ext (4–5 min per confirmed business), although sensitivity was further attenuated (66.7% and 56.3% for Boston and Seattle, respectively).

**Table 4:** Small-areas validation: Cosmetic-procedure business identification and confirmation, sensitivity, and staff time cost by business density comparing various validation methods in Boston and Seattle, 2011–2012 ^a^.

Method	Sub-areas	Number of Cosmetic-Procedure Businesses, by Gold Standard ^b^		Number of Candidate ^c^ Businesses Identified		Number of Businesses Confirmed After Confirmation Procedures ^d^		Sensitivity (%) ^e^		Staff Time Cost ^f^ Per Confirmed Business (in minutes)	
		Boston	Seattle	Boston	Seattle	Boston	Seattle	Boston	Seattle	Boston	Seattle
**Gold-Standard List**	High	37	10								
	Medium	9	5								
	Low	2	1								
	All 3 Areas	48	16								
**1 ^g^**	High			N/A	N/A	17	4	45.9	40.0	3.5	3.5
	Medium			N/A	N/A	1	4	11.1	80.0	3.5	3.5
	Low			N/A	N/A	1	1	50.0	100.0	3.5	3.5
	All 3 Areas			N/A	N/A	19	9	39.6	56.3	3.5	3.5
**2 ^h^**	High			45	2	22	0	59.5	0.0	5.4	N/A
	Medium			3	3	2	2	22.2	40.0	3.8	2.7
	Low			1	1	1	1	50.0	100.0	2.5	1.8
	All 3 Areas			49	6	25	3	52.1	18.8	4.9	3.6
**2 + 2-ext ^i^**	High			89	21	24	6	64.9	60.0	9.3	6.3
	Medium			7	14	3	2	33.3	40.0	5.8	12.6
	Low			5	2	2	1	100.0	100.0	6.3	3.6
	All 3 Areas			101	37	29	9	60.4	56.3	8.7	7.4
**3 ^j^**	High			155	98	37	10	100.0	100.0	10.5	17.6
	Medium			77	29	8	3	88.9	60.0	24.1	17.4
	Low			8	10	2	1	100.0	100.0	10.0	18.0
	All 3 Areas			240	137	47	14	97.9	87.5	12.8	17.6
**1 + 2**	High			N/A	N/A	28	4	75.7	40.0	3.8	4.4
	Medium			N/A	N/A	3	4	33.3	80.0	3.7	4.0
	Low			N/A	N/A	1	1	50.0	100.0	3.5	3.5
	All 3 Areas			N/A	N/A	32	9	66.7	56.3	5.0	4.1
**1 + 2 + 2-ext**	High			N/A	N/A	30	7	81.1	70.0	8.5	6.6
	Medium			N/A	N/A	4	4	44.4	80.0	5.3	8.9
	Low			N/A	N/A	2	1	100.0	100.0	6.8	5.3
	All 3 Areas			N/A	N/A	36	12	75.0	75.0	8.1	7.3

Notes: ^a^ Within each city, three polygons of comparable size were selected based on density of cosmetic-procedure businesses (high, medium, and low). ^b^ Gold-standard list combined results of all three methods (1, 2, 2-ext, and 3) to identify all cosmetic-procedure businesses in a sub-area. ^c^ Candidate businesses are those identified by a validation method using keywords or North American Industrial Classification System (NAICS) codes as a business that possibly offers eligible cosmetic procedure. ^d^ Confirmation procedures involved Internet searches and/or phone calls to determine whether or not a candidate business offered one or more eligible cosmetic procedure and if business located within Boston or Seattle city limits. ^e^ Sensitivity calculated as: Number businesses in an area identified by a method and confirmed through verification / Total number businesses in area as determined by gold standard. ^f^ Staff time cost calculated as: Total minutes of staff time spent to verify candidate businesses identified by a method / Total number of businesses identified by a method and confirmed through verification. ^g^ Method 1: This method used the Internet search tools Google, Yahoo, Yelp, and Bing to identify candidate businesses using predetermined keywords and the city name (e.g., “medspa Boston,” “cosmetic dermatology Boston”). ^h^ Method 2: This method identified candidate businesses using 11 separate keyword searches of ESRI Business Analyst 2011 Boston and Seattle all-business databases.^i^ Method 2-ext: This method extends Method 2 to include candidate businesses identified using a search of four additional keywords using ESRI Business Analyst 2011 Boston and Seattle all-business databases. ^j^ Method 3: This method identified candidate businesses using NAICS codes in a search of ESRI Business Analyst 2011 Boston and Seattle all-business databases. NAICS codes were used for this search if they were associated with confirmed businesses identified in either Method 1 (Internet) or the Method 2 (search with 11 keywords) or in both.

## 5. Discussion and Conclusions

Cosmetic procedures have proliferated rapidly over the past few decades, with over $11 billion spent on cosmetic surgeries and other minimally invasive procedures and another $2.9 billion spent on U.V. indoor tanning in 2012 in the United States alone [16,18] While research interest is increasing in tandem with the growth of the industry, to our knowledge, our study is the first to attempt to develop methods to identify and geographically locate the myriad types of businesses purveying cosmetic procedures in a city. The type of methodological work we undertook is required for rigorous health-linked spatial analyses that have been so important for research in other public health domains, such as tobacco and alcohol use and fast food consumption [60,61,62,63,64]. 

In our pilot study, we found that while locating cosmetic-procedure businesses was challenging, as there is no centralized, uniform system that catalogues these types of businesses nor is there a single or small set of NAICS codes by which to identify them, we were able to identify numerous businesses that offered a range of cosmetic procedures and map their location, which was the primary goal of our study.

Comparing a variety of methods for business identification, we found they varied substantially in terms of both sensitivity and staff time per confirmed business required to achieve a certain level of sensitivity. Methods 1 and 2 required the least staff time to implement, approximately 3.5 min or less per confirmed business; however, these methods also produced low sensitivity relative to our gold-standard list, in which all three approaches were implemented. Method 3 produced the highest sensitivity for both cities (97.9% for Boston, 87.5% for Seattle), but also required high staff time cost (approximately 18 min per confirmed business). Based on our pilot validation study results, we can make the following recommendations: Under conditions where a study is well-resourced, Method 3 would be the strongest approach for maximizing sensitivity in identifying businesses offering cosmetic surgeries, other minimally invasive procedures, and U.V. indoor tanning. Alternatively, under conditions where researchers are working with little support for staff time, our results suggest that the combination of Methods 1, 2, and 2-ext can achieve acceptable sensitivity (75.0%) with moderate staff-time demand. 

Geographic location of these businesses plays an important role in processes by which societal appearance values are transmitted and reinforced. The Tripartite Influence Model suggests the transmission and reinforcement of these values occur through social interaction with peers, family, and media. Yet other research stresses the importance of expanding investigation of the environmental influences on body dissatisfaction and disordered and harmful appearance-control behaviors beyond media. In addition to media, the model should include products and services used for appearance-control and businesses that sell these products and services, namely, cosmetic-procedure businesses. As access to cosmetic procedures is a critical final link once societal appearance values are internalized, geographic location of these businesses is essential knowledge in efforts to estimate the public health impact of this industry.

### 5.1. Limitations

Our pilot study has several important limitations. Resource constraints did not allow us to conduct validation in each whole city, instead only in three areas within each city. To help account for differences across the cityscape, we purposely selected areas of each city that had high, medium, and low densities of cosmetic-procedure businesses so that we could assess the performance of each business-identification method under these different density scenarios. Also, we conducted our pilot study in just two large cities. Other cities across the country in addition to suburban and rural areas will vary in terms of types and volume of cosmetic-procedure businesses, degree of infiltration of cosmetic procedures into other business categories, and percentage of businesses offering cosmetic procedures that advertise these services on the Internet. Also due to limited resources, we did not include cosmetic dentistry in our study; however, this area of specialization should be considered in future studies of the expansion and location of cosmetic-procedure businesses. Searches via the Web were a key tool for verification for all business identification methods we evaluated and especially for Method 1. Our phone verification protocol was conducted in English, but in some areas of country, it may be important to include multiple language capacity in the phone verification phase, such as Spanish, Vietnamese, or other languages. Electronic data sources in our study included ESRI Business Analyst, Google.com, Yahoo.com, and Bing.com; however, additional sources used in prior geospatial research [77,78], such as ReferenceUSA and SuperPages.com, may have identified additional businesses not captured by our sources. Our methods may have missed some businesses that legally offer cosmetic procedures but were not identifiable by any of the approaches we used, namely those businesses that do not advertise their service on the Internet, do not include one of our selected keywords in the business name, and are not registered under one of the 20 NAICS codes we investigated. In addition, our methods were not designed to identify businesses or individuals offering cosmetic procedures illegally. The illegal market in cosmetic procedures, such as injection of industrial-grade liquid silicone, particularly targets low-income, immigrant, and transgender women and is a serious public health concern associated with excessive morbidity and mortality. [38] Other methods will be needed to identify and geographically locate these underground businesses for public health surveillance and legal intervention. 

### 5.2. Conclusions

The cosmetic-procedures industry is increasing in volume and revenues, with the kinds of locations, procedures, and practitioners involved rapidly evolving. While public health concerns are mounting with the growth of the industry, there is still limited, systematic knowledge of what types of procedures are offered, in what types of settings, and in what locations. Our study presents an important first step in developing methods to study the spatial distribution of U.S. businesses offering cosmetic surgery, other minimally invasive procedures, and U.V. indoor tanning. Given the numerous health risks associated with these procedures coupled with the difficulty of identifying cosmetic-procedure businesses, our study highlights the need for a system that catalogues these types of businesses. The methodology developed through this pilot validation study will enable future spatial research to more fully examine the health and healthcare-cost implications of this poorly understood industry.

## Figures and Tables

**Appendix B:**
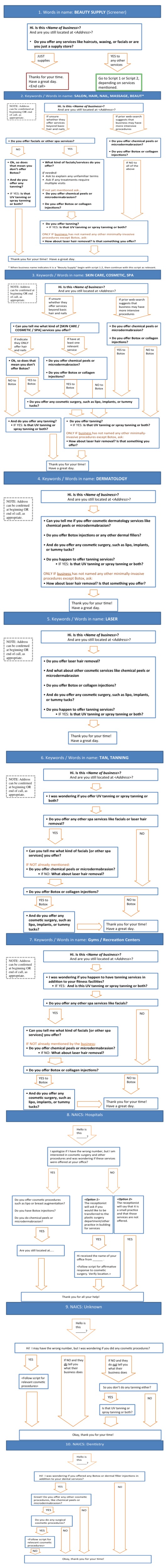
Phone confirmation procedures: Scripts by category of business (defined by keywords in business name or NAICS code).

## Appendix

**Appendix A:** Definitions of cosmetic surgical and minimally invasive procedures from American Society of Plastic Surgeons 2011 (ASPS; http://www.plasticsurgery.org/cosmetic-procedures/).

**Cosmetic Surgical Procedures (Category 1: “Cosmetic Surgery”)**	
Breast “augmentation” (Breast enlargement, augmentation mammoplasty)	Involves using implants to enlarge breasts
Breast implant removals	
Breast lift (Mastopexy)	From ASPS: “Commonly referred to as a breast lift or boob lift, mastopexy surgery raises and firms the breasts by removing excess skin and tightening the surrounding tissue to reshape and support the new breast contour.”
Breast reduction for men (Gynecomastia)	The surgical reduction of enlarged breasts in men.
Buttock implants	
Buttock lift	
Calf enlargement	
Chin surgery (Implant = Mentoplasty)	A surgical procedure to reshape the chin either with implant (enlarge) or with reduction surgery on the bone
Dermabrasion	Dermabrasion and dermaplaning modify the skin’s top layers through a method of controlled surgical scraping. Most often used to improve the look of facial skin left scarred by accidents or previous surgery, or to smooth out fine facial wrinkles. Can be performed on small areas of skin or on the entire face. Most common risk is a change in skin pigmentation (e.g., permanent darkening of the skin due to sun exposure following surgery; or treated skin remains a little lighter or blotchy in appearance). Possibility of excessive scar tissue (keloid or hypertrophic scars); usually treated with the application or injection of steroid medications to soften the scar.
Ear surgery (Otoplasty)	ASPS website text mentions this in relation to both adults and children.
Eyelid surgery (Blepharoplasty)	Modifies eyelids by altering: fatty deposits (“puffiness”) in the upper eyelids; loose or sagging skin; excess skin and fine wrinkles of the lower eyelid; bags under the eyes; and droopiness of the lower eyelids
Facelift (Rhytidectomy)	A surgical procedure designed to reduce: sagging, creases below lower eyelids, creases along nose, jowls, skin under chin (double chin)
Forehead lift (Brow lift)	A surgical procedure designed to reduce creases across the forehead or bridge of nose
Hair replacement (Surgical hair transplants)	Transplant techniques for more modest change include: punch grafts, mini-grafts, micro-grafts, slit grafts, and strip grafts Techniques for more dramatic change include: flaps, tissue-expansion and scalp-reduction
Lip enlargement (not using injectable materials)	
Liposuction (Lipoplasty)	Surgical procedures to remove fat Small incisions are made; cannula (thin, hollow tube) inserted and used to loosen fat; dislodged fat suctioned out using vacuum or syringe.
Lower body lift	Surgical procedure in which sagging skin and fat are removed from lower body
Nose reshaping (Rhinoplasty)	From ASPS: “Surgery of the nose can reduce or augment nasal structures with the use of cartilage grafted from other areas of your body. Most commonly, pieces of cartilage from the septum, the partition in the middle of the nose, is used for this purpose. Occasionally a piece of cartilage from the ear and rarely a section of rib cartilage can be used.”
Pectoral implants	
Thigh lift	Removal of sagging skin and fat
Tummy tuck (Abdominoplasty)	From ASPS: A surgical procedure in which “excess fat, tissue and skin are removed” and “weakened abdominal muscles are repaired and sutured.”
Upper arm lift (Arm lift = brachioplasty)	Removal of skin and fat between underarm and elbow.
**Cosmetic Minimally Invasive Procedures (Categories 2 and 3)**	
Botox, Dysport (#2) (Botulinum Toxin Type A)	In this context, used to reduce facial wrinkles. From ASPS: “Botulinum toxin type A and botulinum toxin type B are derived from a bacteria. Injections of this substance blocks muscular nerve signals, which then weakens the muscle so that it can't contract”
Cellulite treatment (Velosmooth, Endermology)	FDA-approved brands of cellulite treatment: Accent XL, Aluma, Body Jet, Thermage, Tital, TriActive, VelaShape, VelaSmooth According to the Mayo Clinic, lasers and radiofrequency systems may be the most promising treatments on the horizon Mayo also notes that “A twice daily application of 0.3 percent retinol cream has been shown to improve the appearance of cellulite after six months.”
Chemical peel (Chemexfoliation; Derma peeling)	Chemical solutions are applied to skin to improve texture by removing damaged outer layers. Chemicals used are phenol, trichloroacetic acid and alphahydroxy acids. (Diff combos by diff providers) Levels: light, medium, and deep Light: combination of alphahydroxy acids (AHA) and beta hydroxy acids (BHA), such as glycolic acid, lactic acid, salicylic acid and maleic acid (mildest forms) Medium: using trichloroacetic acid (TCA), sometimes used in combination with glycolic acid. Deep: Uses phenol, strongest chemical; may use local anesthetic or sedative; typically requires pre-trt (up to 8 wks before)
Intense Pulsed Light (IPL) treatment	Similar to laser light treatments, but different form of light (range of frequencies, rather than just one freq., like lasers) IPL is a trademarked name; according to Wikipedia, equivalent procedures may go by other terms: VPL, SPL, SPFT, SPTF, SIPL, PTF, CPL, AFT, E-Light, ELOS, M-Light, and others
Laser hair removal	
Laser skin resurfacing (ablative; non-ablative [Fraxel, *etc.*])	Also known as a laser peel, laser vaporization or lasabrasion From ASPS: “laser beam used in laser resurfacing will remove outer layer of skin, called the epidermis. It simultaneously heats the underlying skin, called the dermis. This action works to stimulate growth of new collagen fibers. As the treated area heals, the new skin that forms is smoother and firmer.”
Laser treatment of leg veins	May include surface laser treatments or endovenous techniques (requiring local anesthesia) Surface laser treatment: Sends very strong bursts of light through the skin onto the vein. This makes the vein slowly fade and disappear. Not all skin types and colors can be safely treated with lasers. Endovenous techniques: For very severe varicose veins (now more common than surgery); small tube put into vein, device closes vein through intensive heat treatment.
Microdermabrasion	From ASPS: Use of a minimally abrasive instrument to gently sand the skin. Uses microparticles, or a diamond-tipped wand, to slough off the top layer (epidermis) of skin and stimulate new skin growth.
Sclerotherapy	“The most common treatment for both spider veins and varicose veins. The doctor uses a needle to inject a liquid chemical into the vein. The chemical causes the vein walls to swell, stick together, and seal shut. This stops the flow of blood, and the vein turns into scar tissue. In a few weeks, the vein should fade.” ^1^ Same vein may need to be treated more than once (every 4–6 wks)
Soft tissue fillers (Dermal fillers)	Injectable products designed to increase volume in face and reduce lines or wrinkles; distinct from Botox injections From ASPS, there are 2 categories: temporary (majority) and semi-permanent Semi-permanent: PMMA: Often used for nasolabial folds; can be removed; Brand names: *Artefill, Articol, Metacrill* Temporary: Collagen: Derived from human skin or animals (bovine or porcine); Brand names: *CosmoDerm, Cosmoplast, Zyderm, Zyplast* Hyaluronic acid: From animal sources; Brand names: *Juvederm, Perlane, Prevelle, Restylane, Capitque, Elevess, Puragen* Calcium hydroxylapatite: Heaviest facial filler, used for deepest creases; Brand names: *Radiesse, Radiance* Polyactic acid: Synthetic material that stimulates body’s production of collagen; Brand names: *Sculptra, New-Fill* Human fat (autologous fat): more intensive—must first undergo liposuction to extract fat before injection

Note: ^1^ U.S. Department of Health and Human Services, Office of Women’s Health, 2011: http://www.womenshealth.gov/publications/our-publications/fact-sheet/varicose-spider-veins.html
